# A study on expression recognition based on improved mobilenetV2 network

**DOI:** 10.1038/s41598-024-58736-x

**Published:** 2024-04-07

**Authors:** Qiming Zhu, Hongwei Zhuang, Mi Zhao, Shuangchao Xu, Rui Meng

**Affiliations:** 1 College of Equipment Support and Management, Engineering University of PAP, Xi’an, 710086 China; 2 Basic Education, Engineering University of PAP, Xi’an, 710086 China; 3 College of Military Basic Education, Engineering University of PAP, Xi’an, 710086 China

**Keywords:** Expression recognition, MobileNetV2, Reverse fusion, Attention mechanism, SELU, Information technology, Scientific data

## Abstract

This paper proposes an improved strategy for the MobileNetV2 neural network(I-MobileNetV2) in response to problems such as large parameter quantities in existing deep convolutional neural networks and the shortcomings of the lightweight neural network MobileNetV2 such as easy loss of feature information, poor real-time performance, and low accuracy rate in facial emotion recognition tasks. The network inherits the characteristics of MobilenetV2 depthwise separated convolution, signifying a reduction in computational load while maintaining a lightweight profile. It utilizes a reverse fusion mechanism to retain negative features, which makes the information less likely to be lost. The SELU activation function is used to replace the RELU6 activation function to avoid gradient vanishing. Meanwhile, to improve the feature recognition capability, the channel attention mechanism (Squeeze-and-Excitation Networks (SE-Net)) is integrated into the MobilenetV2 network. Experiments conducted on the facial expression datasets FER2013 and CK + showed that the proposed network model achieved facial expression recognition accuracies of 68.62% and 95.96%, improving upon the MobileNetV2 model by 0.72% and 6.14% respectively, and the parameter count decreased by 83.8%. These results empirically verify the effectiveness of the improvements made to the network model.

## Introduction

Facial expression recognition (FER)^1^ is one of the key research directions in computer vision. It has been found that facial expressions account for more than 55% of the emotional information conveyed by humans^2^, and in populations with relatively poor language skills such as newborns and the elderly, expressions contain even richer information. Currently, FER technology has been widely used in numerous sectors including human–computer interaction^3^, pain identification^4^, fatigue driving judgment^5^, and criminal interrogation^6^.

Facial expression recognition generally includes three steps: preprocessing^7^, feature extraction^8^, and expression classification^9^. Traditional methods for feature extraction implemented designs such as local binary patterns^10^, directional gradient histograms^11^, and scale-invariant feature transformation^12^. However, the above methods are complex in design and feature extraction information is incomplete. At present, researchers have designed a variety of deep learning network models for application in multiple domains such as image processing^13^, natural language processing^14^, and speech recognition^15^, and have achieved good classification results in face expression recognition. Typical models include VGG^16^, RESNET^17^, ALEXNET^18^, GoogLeNet^19^, MobileNetV1^20^, MobileNetV2^20^ MobileNetV3^21^ and other models. In MobileNetV1, the size and computational complexity of the network structure was reduced by replacing the standard convolution with a Depthwise Separable Convolution^22^. on the other hand, MobileNetV2 adds multiple pointwise convolutions while maintaining the depthwise-separable convolutions to further improve the performance of the network structure and reduce the computational complexity. However, when the network structure of MobileNet series is applied to the task of image classification, due to the internal design of the network, optimization strategy and activation function, it will constantly ignore the negative feature information in the input information, which may be the key point of image classification. So, Improvement methods for MobileNet network structure in the field of image classification have been successively proposed in recent years. For example, in 2019, Yonis Gulzar^23^ designed a specific five-layer in mobilenetv2 network while retaining the pre-trained model using migration learning, and achieved good results in automatically extracting fruit features for recognition. Yue Pang^24^ designed an improved mobilenetv2 network in order to solve the problem of sheep recognition and tracking in large-scale sheep farming, while a series of validation tests were carried out. The algorithm utilizes facial features to recognize individual sheep, and the model has the highest accuracy rate among similar algorithms. B. Anil Kumar^25^ et al. added five different layers to the pre-trained MobileNetV2 architecture in order to obtain better classification accuracy with fewer training parameters for a given face detection data. The experimental results show that the method achieves 99.64% recognition accuracy for photo images and higher accuracy for real time video images. Literature^26^ introduced the attention module into the MobileNetV1 model to enhance local feature extraction of facial expressions. Then, the center loss and cross-entropy loss are combined to optimize the model parameters to reduce the intra-class distance and increase the inter-class distance, and the experimental results show the effectiveness of the method.

This paper proposes the following improvements based on previous studies.

(i) Channel attention mechanism with residual network structure is used in the feature extraction stage to extract features from the image.
(ii) In order to retain the negative value information in the features, inverse and fusion operations are performed on the feature map, which is conducted to the next layer by deep fusion convolution to avoid the loss of negative value information when passing through the activation function.
(iii) SELU activation function is used instead of RELU6 activation function for the operation to improve the feature extraction ability of the model.

## Database and model

### Dataset selection

**Figure 1:**
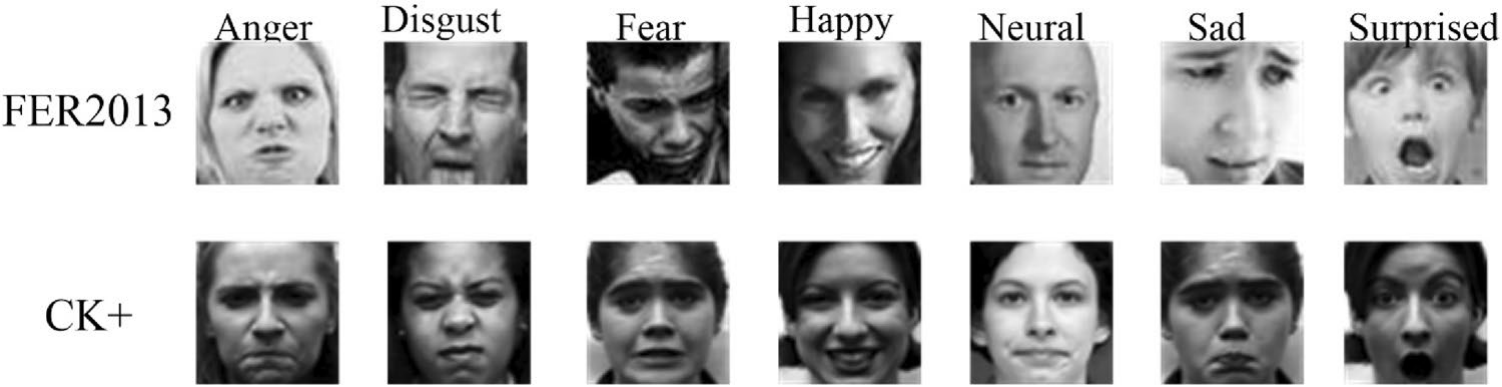
Example of FER2013^28^ and CK + ^29^ images.

The publicly available datasets FER2013 and CK + were used as the datasets used for training and testing. FER2013^27^ Expression Dataset Created by Carrier and Courville for the ICML2013 Facial Expression Recognition Competition. The .FER2013 dataset contains 35,886 face expressions, including face expressions of different age groups in daily life, the pixel values of the images are all 48*48, and they are categorized into seven types of expressions: angry, disgust, fear, happy, sad, surprise and neutral. It is divided into three sections: training, validation and testing. Training section contains 28,709 images, validation section contains 3589 images and testing section contains 3589 images. Each image was labeled with the correct facial expression category. The highest human recognition accuracy on the FER2013 dataset was only 65% to 70% due to acquisition and labeling errors. The CK + ^28^ database is an extension of the Cohn-Kanade Dataset, which consists of seven expressions: anger, tempt, disgust, fear, happy, sadness, and surprise, and contains 123 participants and 593 image sequences. The CK + dataset is a more general face expression dataset, which is suitable for the research of face expression recognition. Figure 1 represents some samples from the FER dataset and the CK + expression dataset.

### Deepwise separable convolution

AlexNet won first place in the 2012 ImageNet competition using Convolutional Neural Networks (CNN) for image classification. Subsequent researchers have designed many more excellent network models, such as VGGNet16/19, GoogLeNet, ResNet50, etc., which have achieved good advantages over traditional classifiers. However, with the deepening of the network, the model computation requires more and more resources, often need to operate on specific machines, which limits the use of deep learning models. Therefore, in 2017, Google Inc. proposed the MobilenetV1 lightweight network model, MobilenetV1 makes full use of the computational resources while maximizing the accuracy of the model in 2018, Google Inc. proposed the mobilenetV2 model based on MobilenetV1.

**Figure 2:**
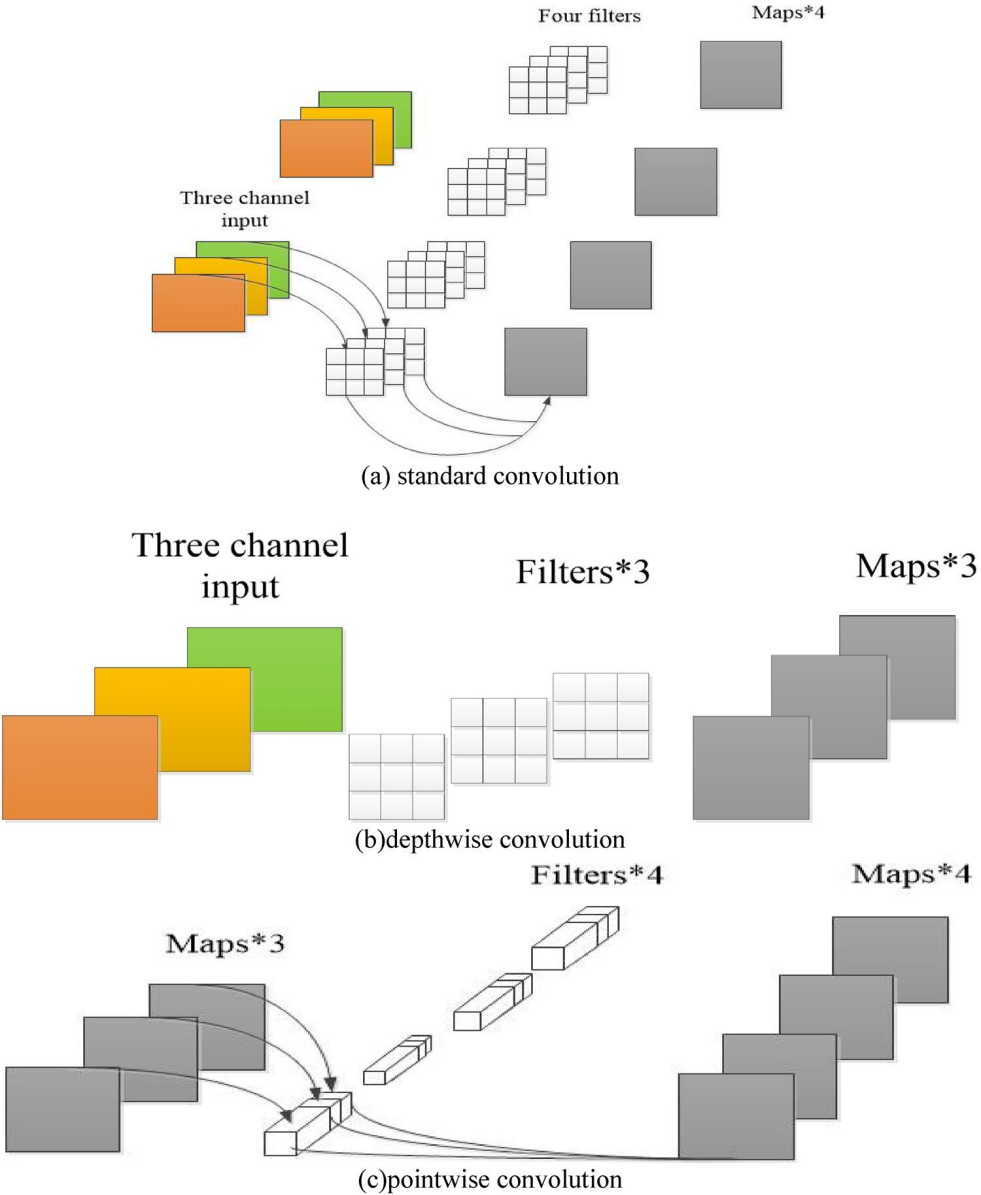
Schematic of the three convolutions.

The traditional standard convolutional operations are characterized as shown in Fig. 2, Suppose the input feature map size is $M \times M$, the number of channels is $C$ ,the convolution kernel size of standard convolution is $N \times N$, the number of $K$. And assuming that the output is the same size as the input, the output size after the standard convolutional kernel is $M \times M$ , and the number of output channels is $K$. Traditional standard convolution actually consists of two steps: filtering the feature map first, combining the filtered results, Fig. 1b shows the standard convolution of the input feature map with the $i(1 \le i \le k)$ convolution kernel. In this process, each channel in the input feature map is first convolved with each channel of the corresponding convolution kernel, The result of the convolution forms a single-channel feature map of C of $M \times M$, and then this C and the result are merged to form a final single feature map of $M \times M \times 1$. Since there are K convolution kernels, the input feature map has a total of K of $M \times M \times 1$ results after standard convolution with all K convolution kernels, The final result is the output feature map of $M \times M \times K$, as shown in Fig. 2a. The standard convolution is computed:

1 $$ M \times M \times N \times N \times C \times K $$

Depthwise separable convolution decomposes the standard convolution into a depth convolution kernel a pointwise convolution, the depthwise convolution process actually convolves each channel of the input with its corresponding convolution kernel individually, and finally the resulting convolution result corresponding to each channel is used as the final depthwise convolution result. In fact, the process of deepwise convolution completes the filtering of the input feature map, and the deepwise convolution process is shown in Fig. 2b, whose computation is:

2 $$ M \times M \times N \times N \times C $$

Here, the pointwise convolution takes the result of depth convolution as input, the convolution kernel size is 1 × 1, and the number of channels is the same as the input. The process of point convolution is similar to standard convolution, which is actually a linear combination of each pixel point on different channels, and retains the original planar structure of the image, regulating the depth. Compared with depth convolution, dot convolution has the ability to change the number of channels, which can accomplish the function of dimension upgrading or downgrading. Dot convolution is shown in Fig. 2c, and its computation is:

3 $$ M \times M \times 1 \times 1 \times C \times K = M \times M \times C \times K $$

The total computation of the deepwise separable convolution is:

4 $$ M \times M \times N \times N \times C + M \times M \times C \times K $$

Deepwise separable convolutions versus traditional standard convolutional computations:

5 $$ \frac{M \times M \times N \times N \times C + M \times M \times C \times K}{{M \times M \times N \times C \times K}} = \frac{1}{K} + \frac{1}{{N^{2} }} $$

From the above equation, it can be seen that the depth separable convolution can effectively reduce the amount of computation, if the network uses 3*3 convolution kernel size for convolution, the depth separable convolution can reduce the amount of computation by 8 to 9 times. Compared with the traditional convolutional computation, this approach is less computationally intensive and can effectively extract features with less loss of accuracy.

The researchers introduced a width factor $\alpha$ into the Mobilenet family of networks. $\alpha$ has the effect of regulating the input and output channels so that the input channel is changed from $M$ to $\alpha M$ and the output channel is changed from $N$ to $\alpha N$. Compared with the standard convolutional layer, the number of parameters is reduced by $\alpha^{2}$. The value of $\alpha$ is taken in the range of (0,1], and it is usually set to 1.0, 0.75, 0.5 or 0.25.

### Reverse fusion methods(RFM)

**Figure 3:**
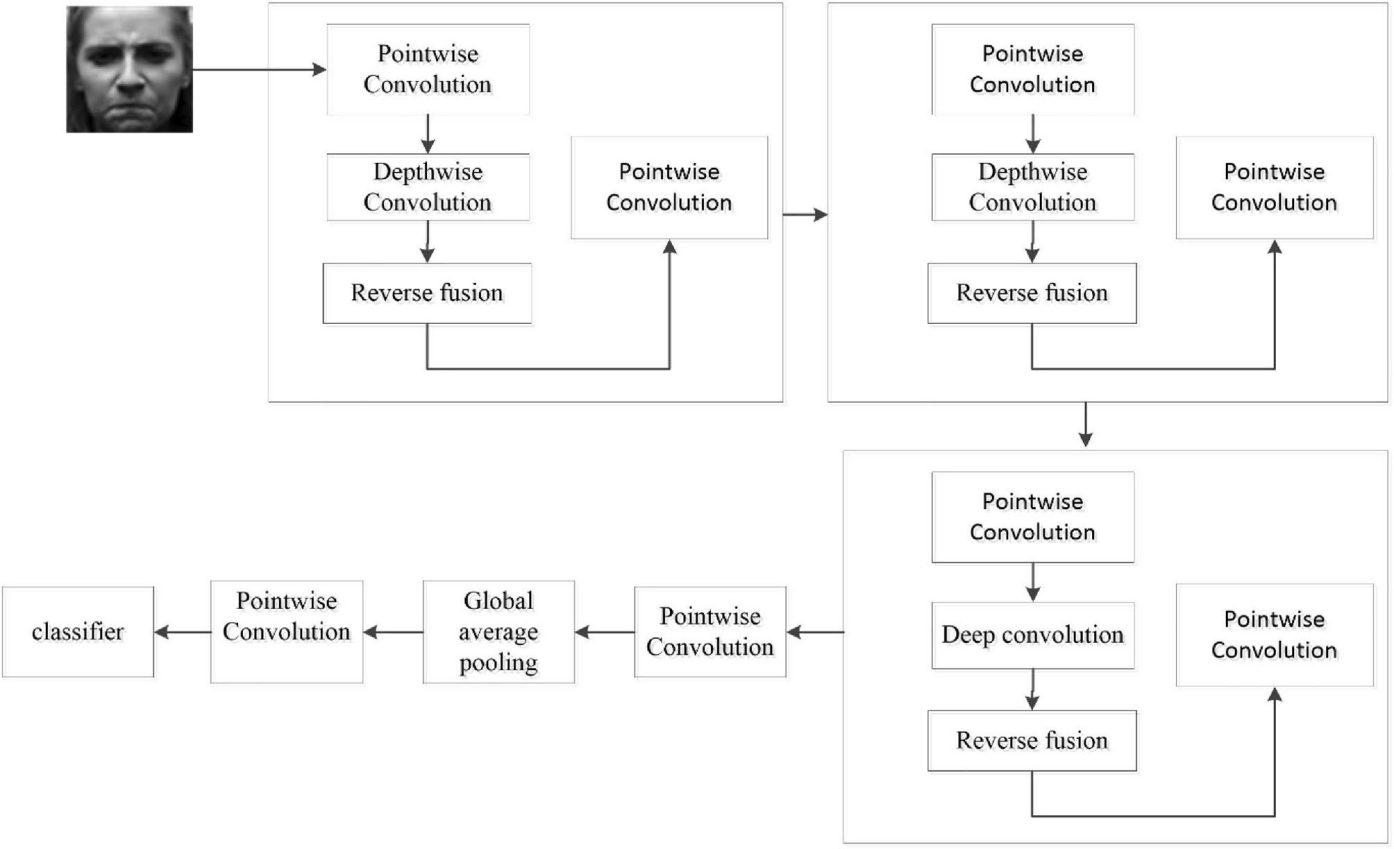
Schematic diagram of reverse fusion operation.

In this paper, we design a deep convolutional inverse layer and a deep convolutional fusion layer to solve the problems of insufficient structural feature extraction and easy to ignore negative feature information in MobileNetV2 network. The original image features and their inverse features are fused and extracted, the class residual structure is added, the convolutional blocks are combined, and the whole network is optimized by loss function for classification. Figure 3 shows a schematic diagram of the reverse fusion operation. The feature map obtained from the deep convolutional layer is passed to the next point-by-point convolutional layer through the deep convolutional inversion layer for the inversion operation, and it is passed to the next convolutional block along with the feature information of the original feature map through the deep convolutional fusion layer for the feature summation, and then to the next point-by-point convolutional layer, and so on. To prevent gradient dispersion, an inverted residual structure is added to the convolution block. The whole network is optimized by cross entropy loss function until convergence. Finally, the global average pooling layer and point-by-point convolutional layer are used to achieve the number of classification requirements, and the input samples are classified by Softmax classifier.

### Attention mechanism

The attentional mechanism is a signal processing mechanism discovered by some scientists in the 1990s while studying human vision. Practitioners in the field of artificial intelligence have introduced this mechanism into some models with success. Nowadays, the attention mechanism has become one of the most widely used “components” in the field of deep learning, especially in the field of natural language processing. The models or structures such as BERT, GPT, Transformer, etc., which have received a lot of exposure in the past two years, all employ attention mechanisms.

**Figure 4:**
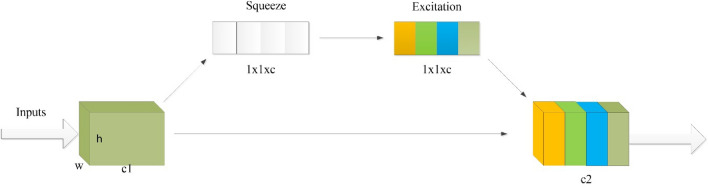
Schematic diagram of the attention mechanism.

Attention mechanisms can be categorized into attention based on spatial relationships and attention based on channel data. The channel-based attention network is to consider the detection target as a single individual, put the addressing target of attention on the addressing of some features of a single individual, and synthesize the feature information of different locations of the individual to give reasonable prediction results. The SE (Squeeze-and-Excitation Networks) attention mechanism is one of the channel attention mechanisms. Essentially, SENET is doing attention on the channel dimension, and this attention mechanism allows the model to pay more attention to the most informative channel features, while suppressing those unimportant or ineffective channel features to train the model for better results. Moreover, the network structure of SENET is relatively simple and can be easily integrated into various neural network structures to improve the performance of the network, and its structure is shown in Fig. 4.

SENET contains a compression operation (Squeeze), an excitation operation (Excitation) and a weight calculation (Scale). For a given input image, the initial extraction of features is accomplished by a convolutional neural network. And then SENET is used for feature weighting calculation, and the calculation process is described as follows, in Eq. 6, *Fsq* denotes global average pooling, while *Uc* denotes the c-th two-dimensional matrix in U and the subscript c denotes channel. $U_{c} (i,j)$ represents the value in the c-th two-dimensional matrix in U, where $1 \le {\text{i}} \le H, \, 1 \le j \le w$.

*H* and *W* denote the height and width of the feature map, and *Uc* denotes the number of channels of the feature map. $\delta$ denotes the relu activation function, $\sigma$ denotes the sigmoid function, $W_{1}$ and $W_{2}$ denote the two fully-connected operations, $A_{{\text{c}}}$ and the weight values of the input features obtained through the SE module denote the outputs obtained through the attention mechanism, $U_{{\text{c}}}^{\prime }$ which are used as inputs for the subsequent computations.

6 $$ Z_{c} = F_{sq} (U_{c} ) = \frac{1}{H \times w}\sum\limits_{i = 1}^{H} {\sum\limits_{j = 1}^{w} {U_{c} (i,j)} } $$

7 $$ A_{{\text{c}}} = F_{ex} (Z_{c} ,W) = \sigma (W_{2} \delta (W_{1} Z_{c} )) $$

8 $$ U_{{\text{c}}}^{\prime } = A_{c} \cdot U_{c} $$

### Activation function

The Relu6 activation function is used in mobilenetv2 network, the relu6 activation function adds a nonlinear constraint to the positive semiaxis of Relu will be greater than six inputs all the output to six to prevent the gradient explosion and the calculation is simple, its expression is as follows:

9 $$ f(x) = \min \;(\max \;(0,x),6) $$

However, relu6 still has the shortcoming that the negative gradient information disappears, and the mean and variance are not 0. In order to improve this problem, the SELU activation function is introduced in this paper.

**Figure 5:**
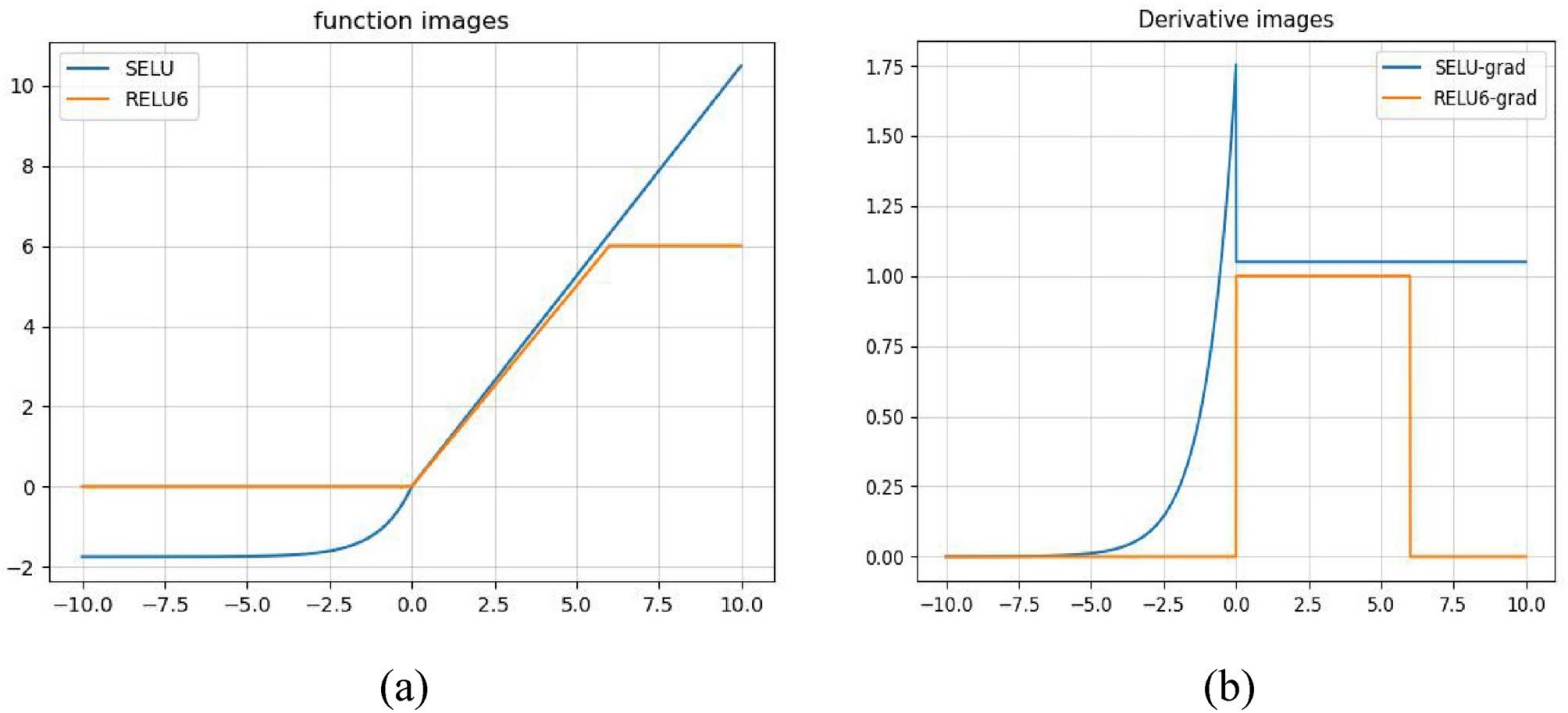
relu6 with SELU function and its derivative image.

SELU allows the input to become a stationary distribution after a certain number of layers, and the positive semiaxis of SELU is greater than 1, which allows it to increase when the variance is too small, but at the same time prevents the gradient from vanishing. So, the activation function then has an immobility point, and the output of each layer when the network is deeper has a mean of 0 and a variance of 1. *β* and *α* are two hyperparameters, and studies have shown that the value of β is usually taken to be 1.05, and the value of α is usually taken to be 1.67. Equation 10 is the SELU function expression. Figure 5 shows the relu6 and SELU function image and its derivative image, where Fig. 5a is a schematic diagram of the SELU function and relu6 function image, when the input is less than 0, the relu6 function output is all 0, at this time, the activation function loses its role, and the SELU function is still able to maintain the negative information, and Fig. 5b is a derivative image of the SELU function and the relu6 function, and when backpropagation is performed When the value is 0, if the value is 0, relu6 gradient information is all 0, the gradient is not being updated, SELU function can still backpropagate and continue to learn the features.

10 $$   f(x) = \beta  \times {\text{ }}\left\{ {\begin{array}{*{20}l}    {\alpha (e^{x}  - 1){\kern 1pt} x < 0} \hfill  \\    {xx \ge 0} \hfill  \\   \end{array} } \right.{\text{ }}   $$

## Improved mobilenetv2 network

**Figure 6:**
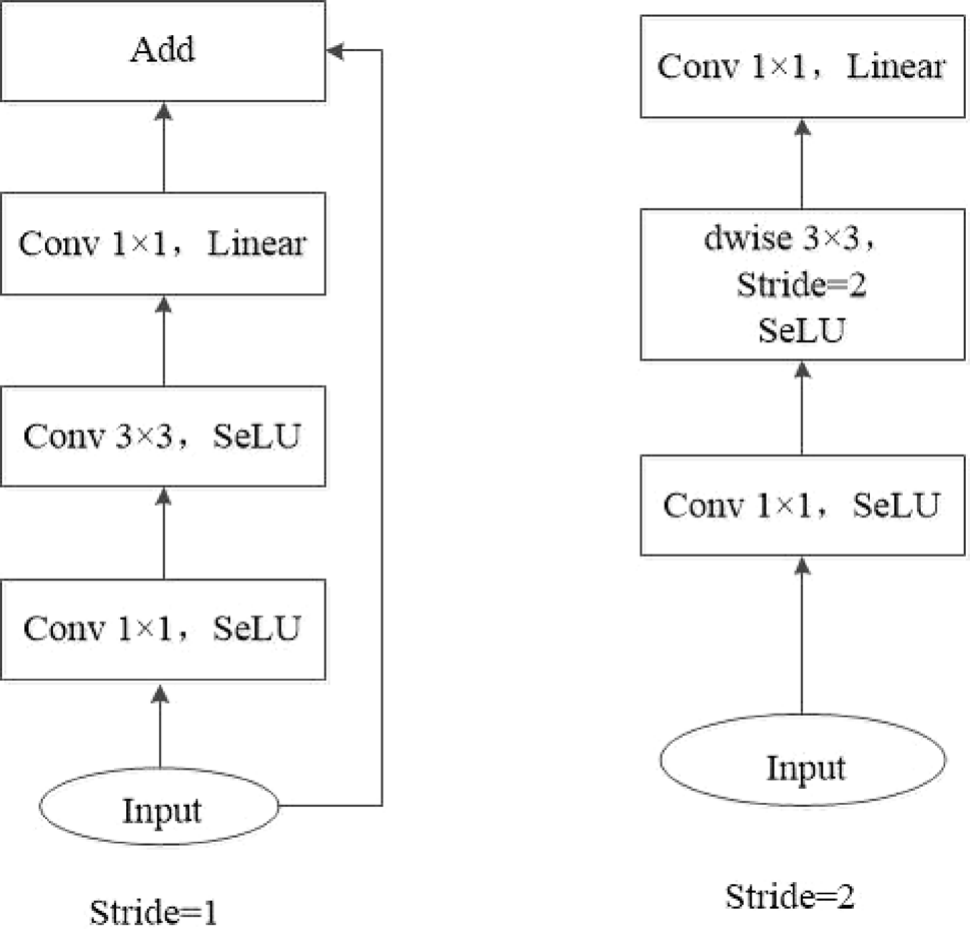
Schematic diagram of inverted residual structure.

The core idea of MobilenetV2 network is to adopt inverted residual structure and linear bottleneck on the basis of MobilenetV1. In order to avoid direct deletion of negative information by the relu6 activation function and to enhance the ability of information extraction, the reverse fusion mechanism and the attention mechanism are integrated into MobilenetV2 network. At the same time, the relu6 activation function is modified to SELU activation function. Figure 6 shows the schematic diagram of the improved inverted residual structure. Table 1 shows the network structure of MobilenetV2.

**Table 1:** MobilenetV2 network structure table.

Number	*t*	Inputs	Stride	*n*	Outputs	Framework	Reverse fusion	SEnet
1	–	3 × 48 × 48	2	6	16 × 24 × 24	Conv2d 3*3		
2	2	16 × 24 × 24	2	2	24 × 12 × 12	block	√	√
3	6	24 × 12 × 12	1	3	32*6*6	block	√	
4	6	32*6*6	1	3	64*3*3	block	√	
5	6	64*3*3	1	4	96*3*3	block	√	
6	6	96*3*3	1	2	160*2*2	block	√	√
7	6	160*2*2	1	3	320*2*2	block	√	
8	–	320*2*2	1	1	320*1*1	Avgpool2d		
9	–	320*1*1	1	1	*k*	Conv2d 1*1		

The input features are first upscaled by a 1*1 convolution, then by a 3*3 convolution, and finally downscaled by a 1*1 convolution layer. Stride denotes the step size, which is used to down sample the feature layer size reduction. Down sampling is only used in the first inverted residual structure of each bottleneck layer (no residual join is used at this point). The inverted residual structure does not use residual connections when stride = 2 or when the input and output channels are not the same. Table 1 shows the network structure table of mobiletv2, where *t* denotes the expansion factor and *n* denotes the number of repetitions.

An image of size 3 × 48 × 48 is input into the network model. The input image first passes through a convolutional layer composed of 3 × 3 convolutional kernels, then through the main framework of the network stacked by the inverted residual module, and then through the global average pooling layer Global Aver-age Pooling(GAP). Finally, a fully connected layer is used to predict the final score of each expression, and the expression with the highest score is the predicted expression. The feature maps are normalized by the Batch Normalization layer after the convolution operation in the convolution layer. Because reducing the width factor can significantly reduce the number of network model parameters and the loss of network accuracy can be controlled. In this paper, we make α = 0.75 and reduce the highest output dimension of the network to 320 , and improve the network accuracy by other improvement measures.

## Experimental method and results analysis

### Experimental environment

The experiments in this paper on PC are based on pytorch1.8.0 deep learning framework and the programming language is python 3.9 on WINDOWS10 64-bit operating system. The hardware platform is Intel(R) Core(TM) i7-8750H CPU @ 2.20 GHz 2.21 GHz. GPU: NVIDA GeForce GTX 1050 Ti, CUDA 12.1.

## Results and discussion

### Analysis of experimental results for the FER2013 dataset

**Figure 7:**
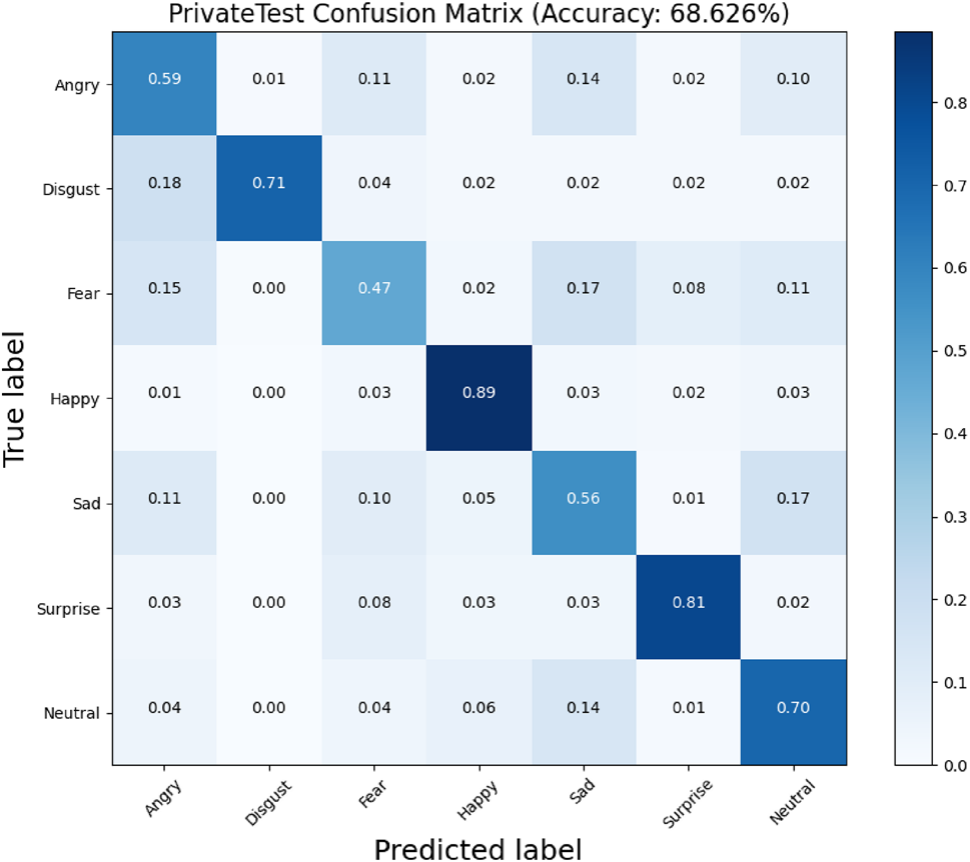
Confusion matrix plot on the FER2013 dataset.

The model is tested on FER2013 dataset, in the experiment the SGD optimizer is used to optimize the loss, the momentum (Momentum) is set to 0.9, the initial learning rate is 0.01, the learning rate weighting factor is 5e-4, epoch is 350, and the batch size is 32. The training set contains a total of 28,709 images, and the public test set (3589 images) is used to adjust the weighting parameters and finally tested on the private test set (3589 images in total).The following Fig. 7 shows the confusion matrix of the network model on the FER2013 dataset.

As can be seen in Fig. 7, the accuracy of the proposed model in this paper is 68.626% on FER dataset. The improvement compared to mobilenetv2 indicates that the feature extraction ability of the model is enhanced by reverse fusion, introduction of the attention mechanism and adoption of the SELU activation function for more accurate recognition. Among them, the recognition rate is higher on happy, surprise, disgust and neutral reaching 89%, 81%, 71%, 70% respectively. Anger, fear, and sadness are prone to misjudgment due to more similarities in their expressions, resulting in lower recognition rates.

### Analysis of experimental results for the CK + dataset

**Figure 8:**
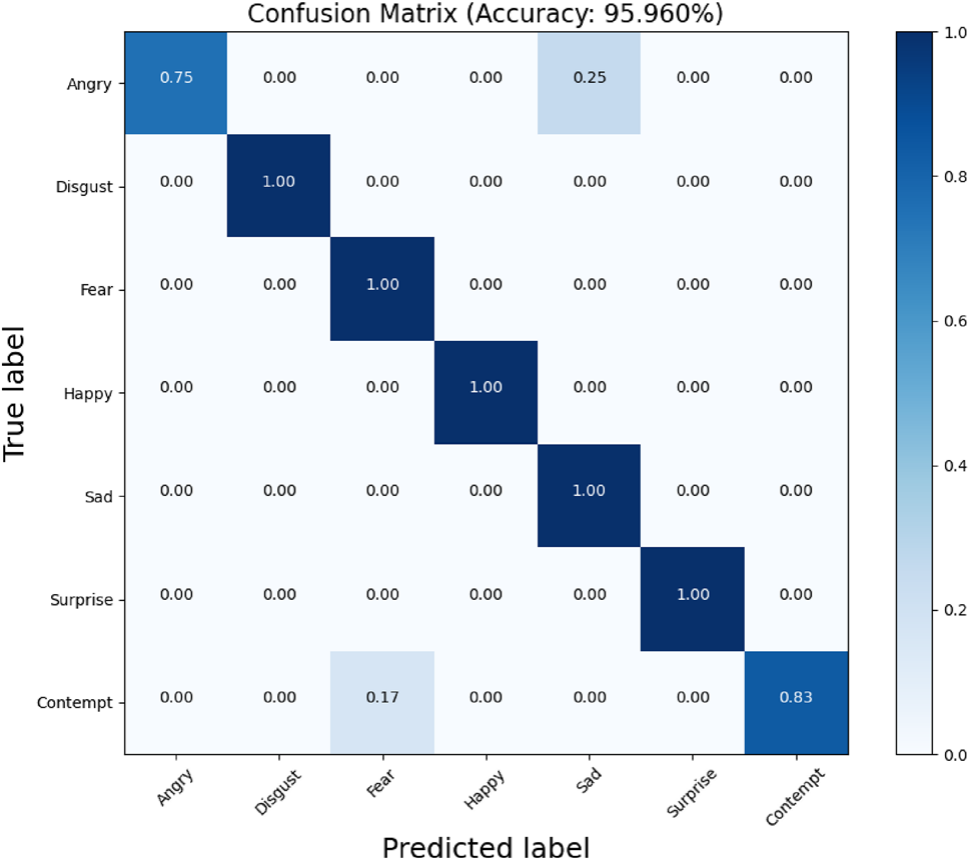
Confusion matrix on the CK + dataset.

The network model in this paper was trained and tested on the CK + dataset, and the weights and biases were randomly initialized during the training process, with a batch size of 20, a number of iterations of 150, an initial learning rate of 0.01, and a stochastic gradient descent optimizer with a momentum factor of 0.9. The model training process was performed using the training set (882 frames) of CK + and tested on the test set (99 frames). The network model is pre-processed by randomly flipping the samples before parameter training on the training dataset. The confusion matrix of the network model on the CK + dataset is shown in Fig. 8.

The model proposed in this paper has an accuracy of 95.960% on the CK + dataset and achieves 100% accuracy on disgust, fear, happy, sad, and surprise, 83% recognition accuracy contempt, and 75% recognition on angry. All have achieved better results. There is a significant improvement relative to mobilenetv2, indicating that the feature extraction ability of the model is enhanced by reverse fusion, introduction of the attention mechanism and adoption of the SELU activation function, and the recognition is more accurate.

### Ablation study

In this paper, methods such as adding attention mechanism, reverse fusion mechanism and SELU activation function are used to strengthen the feature extraction ability of the network, and at the same time, the compression of the width factor is used to reduce the number of model parameters. This paper conducts comparative ablation study on two datasets, FER2013 and CK + to analyze the impact of different improvements in on the experimental results. The number of model parameters in this paper is only 3.26 M, which is only 16.2% of the base model. However, the performance is improved by 0.72% and 5.10% on the two datasets, especially on the CK + dataset. Both the reverse fusion mechanism and the attention module can improve the model performance, the SELU activation function has no effect on the number of model parameters, and the three approaches achieve the effect of reducing the number of parameters and improving the model accuracy when acting on the mobilenetv2 base model. Table 2 shows the comparison of the accuracy of different models using different parts on the dataset.

**Table 2:** Results of ablation experiments comparing FER2013 with CK + dataset.

NET	Params Size/MB	Accuracy rate /%	
		FER2013	CK +
Mobilenetv2	20.10	67.90	89.82
Mobilenetv2(0.75)	3.33	66.99	86.82
Mobilenetv2(0.75) + SE	3.45	67.48	90.91
Mobilenetv2(0.75) + RFF	3.33	68.22	91.92
Ours	3.26	68.62	95.96

### Comparison experiment results and analysis

To compare the advancedness of the proposed network model, this section will compare it with the current mainstream network model. As shown in Table 3, there is a slight difference in accuracy between the network model in this paper and the traditional network model, but there is a significant decrease in the number of parameters, which is only 3.6% and 4.5% of that of VGG19 and Res-Net50. On the FER2013 dataset, the accuracy of the base model used in this paper decreases compared with the traditional deep neural network. On the CK + dataset, the accuracy is equal to that of ResNet50 and 1.32% higher than that of VGG19. Among the lightweight network models, the model in this paper has different degrees of improvement in accuracy and number of parameters, and the improvement is more obvious in CK + . On the FER2013 dataset, the accuracy is improved by 1.06% and 1.46%, and the number of parameters is reduced by 16.84 MB and 18.42 MB compared with the same series of MobileNetV2 and MobileNetV3, respectively. Compared with Xception, a lightweight network, the accuracy increased by 1.7% and the number of parameters decreased by 0.79 MB.On the CK + dataset, the accuracy is improved by 6.14% and 7.09% compared with the same series of Mobile-NetV2 and MobileNetV3. Compared with other lightweight networks, Xception, the accuracy is improved by 3%.

**Table 3:** Accuracy of different models for expression recognition on FER2013 and CK + dataset.

NET	Params Size/MB	Accuracy Rate/%	
		FER2013	CK +
Vgg	89.59	70.30	94.64
Resnet50	72.31	72.86	95.96
Mobilenetv2	20.10	67.90	90.90
Mobilenetv3^29^	21.50	67.50	88.87
Xception^30^	4.35	67.26	92.96
Ours	3.26	68.62	95.96

By comparing with the current mainstream deep convolutional neural networks and lightweight networks on the two datasets, it can be seen that the model in this paper greatly reduces the number of parameters with a small amount of accuracy loss, and the comprehensive performance is higher than that of the current mainstream lightweight networks. The main reasons are.

1. The adjustment of the depthwise separable convolution and width factor makes the number of parameters of the model decrease significantly.
2. The reverse fusion mechanism is introduced to reduce the loss of feature information in the convolutional layer.
3. The SE mechanism is introduced, and the attention mechanism is integrated in the dimension of the channel, which significantly enhances the ability of extracting effective feature information and weakens the extraction of invalid information.

### Real-time testing on mobile

In order to verify the real-time performance of this paper's model on the mobile side, this paper also compares the real-time performance of I-MobileNetV2 with MobileNetV1 and MobileNetV2 models on Huawei p60 cell phone on the mobile side, and selects an image of a typical expression of each of the seven expressions on the CK + dataset to make predictions, and the results of the predictions made by the various models after 1,000 times are shown in Table 4.

**Table 4:** Performance comparison of different models on mobile devices.

model	Average prediction time/ms
MobileNetV1	221
MobileNetV2	300
ours	78

In Table 4, the real-time test of ours network is much better than that of MobileNetV1 and MobileNetV2, indicating that the model in this paper has better real-time performance compared to MobileNetV1 and MobileNetV2 models, and the model in this paper reduces the network parameters by adjusting the width factor, and also improves the real-time performance.

## Conclusion

In this paper, a lightweight facial expression recognition network model with feature fusion channel attention mechanism is proposed to solve the problems of complex structure of traditional convolutional neural network, which leads to excessive parameterization, and insufficient extraction of feature information of face expression. The network model is based on the MobileNetV2 network, the width factor and the overall network dimension are compressed, the feature information is fused, and the efficient channel attention module is embedded to recognize facial expressions. The accuracy of the proposed network model on FER2013 and CK + datasets reaches 68.62% and 95.96% respectively, which is 0.72% and 6.14% higher than that of MobileNetV2, and the number of parameters decreases by 83.79%. The experimental results show that the network model in this paper shows better performance in terms of accuracy and number of parameters compared with many other convolutional neural network models, which verifies the effectiveness of the network model improvement measures. In the subsequent more in-depth research, the lightweight multi-scale convolution can be used for feature extraction and feature fusion to solve the problems of insufficient ability of single-scale convolutional kernel to extract feature information and limited sensory field.

## Data Availability

FER2013 and the “CK + database” are publicly available and can be downloaded at https://www.kaggle.com/datasets/msambare/fer2013. https://www.kaggle.com/datasets/shawon10/ckplus.
